# Bi-specific and tri-specific antibodies- the next big thing in solid tumor therapeutics

**DOI:** 10.1186/s10020-018-0051-4

**Published:** 2018-09-24

**Authors:** Karie Runcie, Daniel R. Budman, Veena John, Nagashree Seetharamu

**Affiliations:** 1 Department of Medicine, Hofstra-Northwell School of Medicine, Hempstead, USA; 2 Division of Hematology and Medical Oncology, Hofstra-Northwell School of Medicine, Hempstead, USA

**Keywords:** Bispecific antibody, Trispecific antibody, Immunotherapy, Solid tumor

## Abstract

Antibody-based therapy has revitalized the world of cancer therapeutics since rituximab was first approved for the treatment of Non-Hodgkin’s Lymphoma. Monoclonal antibodies against cancer antigens have been successful strategies for only a handful of cancer types due to many reasons including lack of antibody specificity and complex nature of tumor milieu which interfere with antibody efficacy. Polyspecific antibodies are promising class of anti-cancer agents which can be directed at multiple tumor antigens to eradicate tumor cells more precisely and effectively. They may overcome some of these limitations and have already changed treatment landscape for some malignancies such as B cell acute lymphoblastic leukemia. Pre-clinical studies and early phase clinical trials have demonstrated that this approach may be an effective strategy even for solid tumors. This review focuses on the development of bispecific and trispecific antibody therapy for the treatment of solid tumor malignancies and highlights the potential they hold for future therapies to come.

## Background

Cancer remains the second leading cause of death in the United States, with lung cancer being the leading cause of cancer deaths, followed by breast cancer in women and prostate cancer in men (Siegel 2017). Over the past few decades, new and targeted therapies have contributed to significant improvements in the 5-year relative overall survival rate for all cancers combined, most prominent in hematopoietic and lymphoid malignancies (Siegel 2017). An integral part of this revolution has been development of monoclonal antibodies in the 1970s. Rituximab, a genetically engineered chimeric antibody against the CD 20 antigen found on the surface of B cells, was the first monoclonal antibody approved by the Food and Drug Administration in 1997 for the treatment of Non-Hodgkin’s lymphoma (Leget and Czuczman 1998; White et al. 2000). Since then, the use of monoclonal antibodies for cancer therapy has evolved to target different molecules and has expanded treatment options for solid tumors as well as hematologic malignancies. Despite the excellent tolerability profile and efficacy in various cancers, these agents are rarely effective as monotherapies and have several limitations. Activation of alternate molecular pathways, acquired changes in the tumor milieu, and suboptimal engagement of monoclonal antibodies to their targets are just some of the drawbacks (Fousek and Ahmed 2015). Recent innovations based upon improvements in protein engineering have resulted in combining antibodies for synergistic effect (Henricks et al. 2015; Binyamin et al. 2006); development of antibodies with genetically engineered Fc receptors to achieve higher effector functions such as antibody-dependent cytotoxicity (Binyamin et al. 2006); and creation of bi-specific and tri-specific antibodies which are capable of binding to multiple targets (Segal et al. 1999; Mertens et al. 2001). A search of Pub-Med in February 2018 reveals 2744 articles on bi-specific antibodies with 1784 mentioning cancer. In an analogous fashion for tri-specific antibodies, a search noted 37 articles with 25 mentioning cancer.

## Historical review

The concept of using a molecule with more than one binding site to enhance its biological function actually dates back to 1961 when two antigen-binding fragments from different polyclonal sera were combined to form bispecific molecules (Nisonoff and Rivers 1961). The techniques of chemical conjugation of two different antigen-specific monoclonal antibodies and fusion of two antibody producing hybridoma cell lines (quadromas) in the 1970s and 1980s took the production of bispecific antibodies to the next level (Staerz et al. 1985; Karpovsky et al. 1984; Perez et al. 1985)**.** Although some of these primitive formats showed appreciable activity against certain malignancies, the vast majority had a dismal therapeutic-risk index. With rapid advances in genetic engineering, the past two decades have seen a dramatic increase in the production of polyspecific antibodies with more than 120 described formats now in clinical use or undergoing evaluation in clinical trials (Spiess et al. 2015). Even though majority of the early development of polyspecific antibodies was focused on hematological malignancies, there are several molecules in clinical development that are directed towards non-hematological cancers. Our review focuses on clinical development of bi-specific and tri-specific antibodies directed towards solid tumors.

## Advantages and disadvantages of polyspecific monoclonal antibodies

**Fig. 1:**
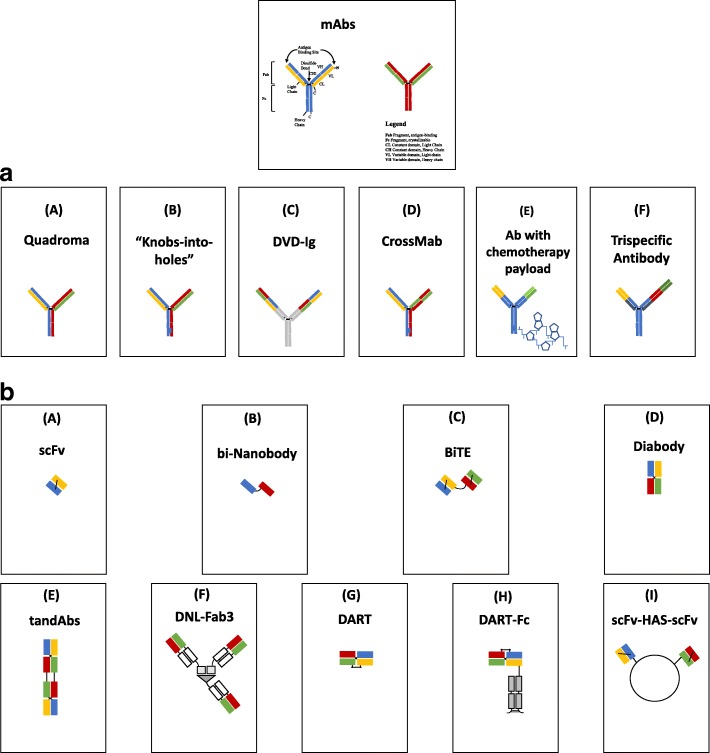
**a**: Some formats of IgG –like bispecific antibodies and a trispecific antibody. (**A**) Quadroma created by combining the light and heavy chains of two different monoclonal antibodies resulting in two antigen binding sites aimed at different tumor antigens eg. Catumaxomab (**B**) Two antibody chains with engineered CH3 domain to create a “knob” in one heavy chain and a “hole” in the other to promote heterodimerization. This allows only specific pairing of heavy chains to reduce mispairing. (**C**) Variable domains of two mABs fused to create a dual- specific antibody (**D**) CrossMab exchanging of the CH1 domain of one heavy chain with the constant domain of the corresponding light chain for better light chain pairing (**E**) Bispecific antibody with two unique antigen-binding sites and a chemotherapy payload attached to the constant domain (**F**) Trispecific antibody engineered to bind to three different ligands on tumor cell. **b**: Some formats of bispecific antibody fragments and fusion proteins. (**A**) scFvs – Combination of the variable region of one light chain with the variable region of one heavy chain fragment is the basic element for antigen binding. (**B**) bi-Nanobody- Combination of two different single variable heavy chain domains which are able to bind different tumor antigens. (**C**) BiTE- Tandem single chain variable fragments from different antibodies joined by a flexible peptide chain. (**D**) Diabody – Bivalent molecules composed of two chains each comprising a variable light chain and variable heavy chain domain, either from the same or different antibodies. (**E**) TandAbs - Two pairs of variable light chain and variable heavy chain domains are connected in a single polypeptide chain to form a tetravalent tandAb. (**F**) DNL-Fab3 – Trivalent bispecific antibody composed of three Fab fragments joined by the utilization of the specific interaction between the regulatory subunits of cyclic adenosine monophosphate (cAMP)-dependent protein kinase A (PKA) and the anchoring domains of A kinase anchoring proteins (AKAP). (**G**) DART – Two polypeptide chains derived from the variable heavy chain from one molecule linked to a variable light chain of another molecule. (**H**) DART-Fc - DARTs with a Fc fragment designed to prolong serum retention time. (**I**) scFv-HAS-scFV- Association of two single chain variable fragments through modified dimerization domains

**Fig. 2:**
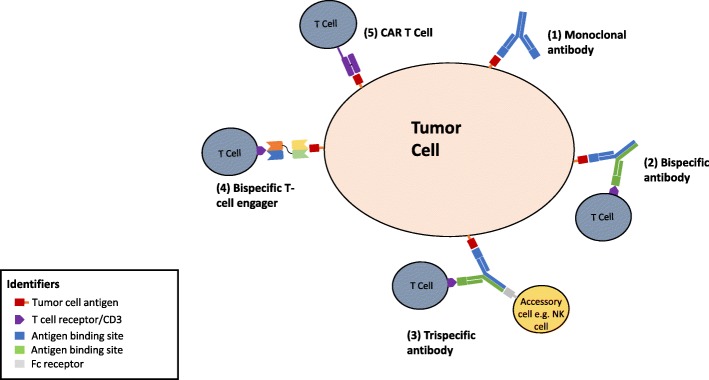
Schematic showing different formats of immunotherapy binding to the antigen on the tumor cell: (1) monoclonal antibody, (2) bispecific antibody, (3) trispecific antibody, (4) bispecific T cell engager made from two single chain antibody fragments, and (5) CAR T cell therapy which is comprised of light chain and heavy chain variable fragments joined by a flexible linker

Polyspecific monoclonal antibodies (PsMabs) are genetically engineered proteins that can simultaneously engage two or more different types of epitopes (Figs. 1a, b and 2) (Fan et al. 2015; Zhang et al. 2017). They show several advantages over monoclonal antibodies (Fig. 2) in that they can: 1) redirect specific polyclonal immune cells such as T cells and NK cells to tumor cells to enhance tumor killing, 2) simultaneously block two different pathways with unique or overlapping functions in pathogenesis, 3) potentially increase binding specificity by interacting with two different cell surface antigens instead of one, and 4) reduce cost in terms of development and production when compared to multiple single based antibodies used in combination therapy or compared to the production of CAR-T cells. The advantages and disadvantages of polyspecific antibodies are tabulated in Table 1.

**Table 1:** Advantages and disadvantages of polyspecific antibodies over monospecific antibodies

Advantages	Disadvantages
Amenable for large scale production	Hetero-dimerization of chains may make the molecule inefficient; early methods had low production yields
More efficient binding to target	Steric inhibition of engaging sites
Able to engage T cell or NK cells (MHC agnostic) by a cell combining site	Potential antigenic cytokine release syndrome
Stability	Small molecules can be rapidly cleared; larger ones may aggregate; potential immunogenicity
Not patient specific; target specific	Tight white cell binding may change bio-distribution
Can be a carrier of radioisotope or chemotherapy	Potential poor internalization of molecule if combined with cytotoxic agent
Can be used for imaging	Need for external epitope
Can serve as an immune enhancer	Affinity for target epitope and effector cell critical
Can be encapsulated in a liposome	Large molecules have less intra-tumoral penetration.
Can be combined with other immunological agents	May enhance toxicity if combined with classical immunological agents
Bystander effect	

## PsMab formats

Bispecific antibodies with ability to engage two different antigens are the most commonly used PsMabs. Since the initial experiments to produce BsMabs (Holliger and Winter 1993), the products have undergone major transformation (Brinkmann and Kontermann 2017) and have proved useful in cancer diagnosis, imaging, and treatment. The differences in the first generation BsMabs and the newer molecules are tabulated in Table 2. The components of polyspecific antibodies used today range from full antibody structures to fragments, with creation of structures only limited by the vision of the molecular chemist, patenting rules, physiochemical constraints, and clinical utility. Some of the BsMab manufactured today include bispecific small molecule-antibody conjugates, chemical hetero-conjugates, and protein genetic engineering involving recombinant DNA technology (Spiess et al. 2015; Brinkmann and Kontermann 2017). The most studied structures are shown in Fig. 1a and b. In general, these bispecific antibodies are divided into two major classes: IgG like bispecific antibodies which carry an Fc region and therefore retain Fc-mediated effector functions and the non-IgG like formats which rely entirely on their antigen binding capacity to exert therapeutic effects. The differences between these two classes are highlighted in Table 3. Fc mediated effector functions include antibody-dependent cell mediated toxicity (ADCC), antibody dependent cellular phagocytosis (ADCP), complement-dependent cyototoxicity, and the binding of the molecules to the neonatal Fc receptor (FcRn) which protects IgG from degradation and increases the molecular half-life (Fan et al. 2015).

**Table 2:** Production, limitations, and examples of first and subsequent generations of BsMab

EarlierBsMab	NewerBsMab
Produced by oxidative recombination, chemical cross-linking, and enzymatic digestion of desired antibodies to yield Fab fragments which are then combined via bifunctional reagents to form a heterodimer^21,22^.	Produced by advanced techniques such as controlled Fab-arm exchange (cFAE), improvised somatic fusion of two hybridoma cell lines (quadroma), small molecule-antibody conjugation, genetic engineering using molecular cloning technology^20^.
Inability to produce large quantities	Shorter processing time and ability to produce in large-scale.
Rapid destruction of murine antibody fragments	Stability and longer half-life.
Difficult to purify	> 90% pure
Examples: MDX-210^23^ (targeting Her2, and CD 64 or FcyRI which is expressed on monocytes, macrophages and activated neutrophils; MDX-447 ^24^(targeting EGFR and CD 64; HRS-3/49- Targeting CD 30 on Reed Sternberg cells in Hodgkin lymphoma and FcyRIII or CD 16 on natural killer cells and macrophages.	Examples: Catumaxomab and Solitomab (targeting EpCAM expressed on breast, ovarian and other cancer cells as well as CD3 on T cells-bispecific T cell engager or BiTE)^23,26,27^; Blinatumomab^28^(binding to both CD19 on B cell cells and CD3 on T cells).

**Table 3:** Comparing different formats of bispecific antibodies

Antibody format	Advantages	Disadvantages
IgG- like bispecifc antibodies	Longer half life	Fc region more immunogenic
	Fc domain facilitates efficient purification	
	Fc domain able to trigger antibody dependent cell mediated toxicity, complement dependent cell mediated toxicity	
Bispecific Antibody Fragments	High tumor specificity	Small size make them prone to elimination i.e. shorter half-life
	Good tissue penetration	
	Small formats can allow close contacts between effector and target cells forming efficient immune synapses not requiring co-stimulatory molecules	
	Easy to manufacture	

In the past, IgG-like BsAbs were created by chemical conjugation or hybridoma techniques. These were cumbersome, time-consuming procedures and resulted in creation of nonfunctional molecules through random assembly of different heavy and light chains in addition to intended bispecific antibodies (Staerz et al. 1985). These shortcomings can be improved using recombinant DNA technology. For example, bispecific tetravalent molecules such as dual-variable-domain immunoglobulin (DVD-Ig) can now be produced by combining two target-binding monoclonal antibodies via naturally occurring linkers and optimizing yield of viable molecules through advanced recombinant DNA techniques (Spiess et al. 2015). The simultaneous binding of antigen to all variable domains in dual variable domain immunoglobulin (DVD-Ig) provide a higher specific binding capacity which could be used in targeting low abundance proteins such as cytokines (Sedykh et al. 2018) and potentially also allow the drug to be dosed less frequently.

Recombinant techniques have also led to the creation of small fragment molecules by combining single chain variable fragments from two different monoclonal antibodies to form bivalent bispecific “antibodies” ranging in size from 50 to 60 kDa (Kontermann 2012)**.** Some examples of these are the bispecific T cell engager (BiTE), tandem single chain variable fragments (taFvs), diabodies (Dbs), single chain diabodies (scDbs), and triplebodies. Due to their small size, these scFV based antibody fragments have high tumor specificity and tumor penetration. However, their small size also limits serum half-lives which could potentially limit efficacy and increase cost by requiring repetitive dosing (Zhang et al. 2017). By fusing these antibody fragments with albumin or proteins that bind albumin, the serum half-life can be prolonged by five or six times (Sedykh et al. 2018)**.** Other methods such as PEGylation, Fc fragment fusion, and multimerization are also being explored to extend antibody serum half-life (Fan et al. 2015). The major advantages and disadvantages of IgG – like bispecific antibodies and bispecific antibody fragments are listed in Table 3.

The BiTE warrants special mention as one of the binding sites is engineered to bring an effector cell (T-cell or NK cell) into the proximity of the tumor cell to enhance antitumor effect (Fig. 2). This function results in more effective tumor cell lysis relative to other bispecific formats and monoclonal antibodies (Yang et al. 2016)**.** In the case of T-cells, the target site for binding is commonly CD3. The bi-specific binding of a white cell to the target mimics the action for an activated white cell against its target (Offner et al. 2006). This functionality of bispecific antibody which can use non-HLA restricted white cells without in vitro expansion was described in 1985 but could not be exploited until recently (Staerz et al. 1985). Another advantage of this approach is a bystander effect on adjacent cells which may be of value in a tumor with heterogeneous expression of target epitope (Ross et al. 2017). It was recently noted in a study looking at the bio-distribution of a T-cell dependent bi-specific antibody in a murine model of human HER2 breast cancer that the binding affinity for the white cell can determine whether or not the bi-specific antibody/white cell combination can reach the target site or becomes trapped in lymphatic organs (Mandikian et al. 2018). Hence, negative studies with bi-specific antibodies may be in part due to lack of target cell engagement due to poor distribution.

The diabody format which has two variable domains of two different antibodies connected by two linkers has more stability than BiTEs but the linkers restrict the mobility of the antigen binding sites limiting antigen recognition (Fan et al. 2015)**.** DART bispecific antibodies, created by engineering two Fv fragments with the variable heavy chain portion exchanged with one another, are larger than BITEs and show better serum stability (Zhang et al. 2017). The Dock-and-Lock (DNL) recombinant method (Fig. 1b) creates multivalency and multifunctionality with bispecific antibody fragments (Rossi et al. 2006)**.** These later generation formats such as BiTE, dual-affinity re-targeting antibodies (DART) antibodies, tandem diabodies appear to potently eliminate targets expressing their tumor associated antigen (TAA) in the absence of costimulatory models or IL-2 pre-activated T cells (Zhukovsky et al. 2016).

## PsMab in clinical use

Currently, there is only one bi-specific antibody approved for cancer therapy. The first bispecific antibody to enter clinic, Catumaxomab was approved by European Union for use in malignant ascites in 2009 (Seimetz 2009). Catumaxomab is a trifunctional antibody produced by a rat-mouse quadroma cell with affinities to CD3 antigen on cytotoxic T cells and epithelial cell adhesion molecule (EpCAM) which is a type 1 transmembrane glycoprotein associated with malignant ascites and effusions and expressed on the majority of epithelial cancers (Linke et al. 2010; Seimetz 2011). The approval was based on studies showing the efficacy of intraperitoneal administration of Catumaxomab in improving symptoms and signs of malignant ascites and reducing the need for paracentesis (Heiss et al. 2010; Frampton 2012). A positive trend of overall survival in patients with malignant ascites associated with epithelial cancers was also noted in a prospective randomized trial (Heiss et al. 2010; Frampton 2012) and there was demonstrable activity in platinum refractory epithelial ovarian cancer (Baumann et al. 2011). The drug was deemed safe for use in outpatient setting in patients with malignant ascites secondary to gynecological tumors including epithelial ovarian cancer and metastatic breast cancer (Kurbacher et al. 2015). Although Catumaxomab showed promising results, it was taken off the market in 2014 due to financial reasons. Its approval was subsequently withdrawn in 2017 (No Title. https://neovii.com/neovii-completes-marketing-authorisation-withdrawal-of-removab-in-the-european-union/ n.d.).

The bi-specific antibody Blinatumomab was approved by the US FDA in December 2014 for the treatment of acute B-cell acute lymphoblastic leukemia (ALL). Blinatumomab is a novel bi-specific T cell engager which binds sites for both CD19 (antigen expressed on all stages of B cell lineage) and CD3 T cell receptor complex, leading to T cell proliferation and activation resulting in target cell (lymphoblast) apoptosis. Unlike catumaxomab, which uses large IgG-like bi-specific antibodies with Fc regions, blinatumomab was created by the fusion of two single chain variable fragments (scFv) connected in a flexible manner through a peptide linker (Newman and Benani 2016). It has been shown to induce durable responses in patients with B cell malignancies and was quickly approved after phase II trial of adult patients with Philadelphia-chromosome-negative relapsed or refractory B-precursor ALL, 43% (81 out of 189) of patients reached the primary endpoint of complete hematological response (Topp et al. 2014). A subsequent multi-institutional phase 3 trial evaluated blinatumomab to standard-of-care chemotherapy in heavily pre-treated B-cell precursor ALL confirmed excellent activity of the drug in these patients with improved outcomes, including overall and progression-free-survivals (Kantarjian et al. 2017).

## Other agents including those in development

The clinical success of these engineered antibodies has ushered in a phase of rapid development of new agents for the treatment of various solid tumor malignancies. Many tyrosine kinase receptors which are integral in regulating the pathway of cell growth, cell differentiation, cell migration, and cell death are prime targets for most of these molecules (Yu et al. 2017; Kalyankrishna and Grandis 2006; Loibl and Gianni 2017; Ooi et al. 2004; Shinojima et al. 2003). A few examples are highlighted here and a detailed log of ongoing clinical trials is presented in Table 4.

**Table 4:** Recent clinical trials involving the use of bispecific antibodies in solid tumor malignancies

Drug name	Antibody type	Sponsor	Target antigens	Development stage	Indication	Status	Clinical trials identifier
Selicrelumab Vanucizumab Bevacizumab	CrossMab	Hoffmann-La Roche	Ang2, VEGF	Phase I	Advanced/ Metastatic Solid Tumors	Recruiting	NCT02665416
rM28, autologous PBMCs	Tandem ScFv	University Hospital Tuebingen	CD28, HMV-MMA	Phase I/Phase II	Stage III/IV metastatic melanoma	Completed	NCT00204594
Indium labeled IMP-205xm734	IgG type bispecific antibody	Radboud University	CEA	Phase I	Colorectal carcinoma	Completed	NCT00185081
Obinutuzumab, RO6958688	IgG type T cell bispecific antibody	Hoffmann-La Roche	CEA, CD3	Phase I	Locally advanced or Metastatic Solid Tumors	Active, not recruiting	NCT02324257
MEDI 565	BITE	MedImmune LLC	CEA, CD3	Phase I	Gastrointestinal Adenocarcinomas	Completed	NCT01284231
Anti-CEA x anti-DTPA	Fusion of two Fab fragments	Nantes University Hospital	CEA, DTPA	Phase II	Medullary Thyroid Carcinoma	Completed	NCT00467506
anti-CEA x anti-HSG TF-2	Dock and lock bispecific antibody	Garden State Cancer Center at the Center for Molecular Medicine and Immunology	CEA, HSG	Phase I	Detection of Colorectal Carcinoma	Unknown	NCT00895323
TF2 antibody/68Ga-IMP-288	Gallium labeled Dock and lock bispecific antibody	Nantes University Hospital	CEA, HSG	Phase II	Metastatic Colorectal Cancer	Completed	NCT02587247
TF2–68 Ga-IMP-288	Gallium labeled Dock and lock bispecific antibody	Nantes University Hospital	CEA, HSG	Phase I/Phase II	HER2 negative Breast Carcinoma expressing CEA	Active, not recruiting	NCT01730612
Anti- CEA x Anti-HSG TF2, Radiation	Dock and lock bispecific antibody	Centre René Gauducheau	CEA, HSG	Phase I/Phase II	Small Cell Lung Cancer CEA-Expressing NSCLC	Completed	NCT01221675
TF2 and 68 Ga- IMP-288	Gallium labeled Dock and lock bispecific antibody	Nantes University Hospital	CEA, HSG	Phase I/ Phase II	Medullary Thyroid Carcinoma	Completed	NCT01730638
TF-2, IMP-288 labeled with In111 and Lu177	Dock and lock bispecific antibody	Radboud University	CEA, Lu177- labeled peptide	Phase I	Colorectal cancer	Completed	NCT00860860
AMG 757	BITE	Amgen	DLL3	Phase I	Small Cell Lung Cancer	Recruiting	NCT03319940
NOV1501	IgG type bispecific antibody	National OncoVenture	DLL4, VEGF	Phase I	Advanced Solid Tumors	Recruiting	NCT03292783
OMP-305B83	IgG type bispecific monoclonal antibody	OncoMed Pharmaceuticals	DLL4, VEGF	Phase I	Metastatic Colorectal Cancer	Recruiting	NCT03035253
OMP-305B83	IgG type bispecific monoclonal antibody	OncoMed Pharmaceuticals	DLL4, VEGF	Phase I	Previously Treated Solid Tumors	Active, not recruiting	NCT02298387
OMP-305B83, Paclitaxel	IgG type bispecific monoclonal antibody	OncoMed Pharmaceuticals	DLL4, VEGF	Phase I	Ovarian, Peritoneal or Fallopian Tube Cancer	Recruiting	NCT03030287
EEDVsMit	Nanocell coated with IgG type bispecific antibody and mitoxantrone payload	Dr. David Ziegler	EGFR	Phase I	Refractory solid or CNS tumors expressing EGFR	Recruiting	NCT02687386
EGFR (V)- EDV- Dox	Nanocell coated with IgG type bispecific antibody and doxorubicin payload	Engeneic Pty Limited	EGFR	Phase I	Recurrent Glioblastoma Multiforme	Recruiting	NCT02766699
TargomiRs	Nanocell coated with IgG type bispecific antibody containing microRNA	Asbestos Disease Research Foundation	EGFR	Phase I	Recurrent malignant pleural mesothelioma Non-small cell lung cancer	Completed	NCT02369198
MDX447	IgG type bispecific antibody	Dartmouth- Hitchcock Medical Center	EGFR	Phase I	Brain and Central Nervous System Tumors	Completed	NCT00005813
Anti-CD3x anti- EGFR bispecific armed activated T cells (BATs), Aldesleukin, Sargramostim	T cells preloaded with IgG type bispecific antibody	Barbara Ann Karmanos Cancer Institute	EGFR, CD3	Phase I/ Phase II	Locally advanced, metastatic, or recurrent pancreatic cancer	Active, not recruiting	NCT02620865
EGFR BATs	T cells preloaded with IgG type bispecific antibody	University of Virginia	EGFR, CD3	Phase I/ Phase II	Locally advanced and metastatic pancreatic cancer	Recruiting	NCT03269526
Anti-CD3 x Anti-EGFR BATs with radiation and temozolomide	T cells preloaded with IgG type bispecific antibody	University of Virginia	EGFR, CD3	Phase I	Glioblastoma Multiforme	Recruiting	NCT03344250
JNJ-61186372	IgG1 type bispecific antibody	Janssen Research and Development LLC	EGFR, cMet	Phase I	Advanced non-small cell lung cancer	Recruiting	NCT02609776
MCLA-158	IgG1 bispecific antibody	Merus N.V.	EGFR, LGR5	Phase I	Metastatic Colorectal Cancer and select advanced solid tumors	Recruiting	NCT03526835
Catumaxomab	Trifunctional IgG type antibody	AGO Study Group	EpCAM, CD3	Phase II	Ovarian cancer, Fallopian Tube Neoplasms, Peritoneal Neoplasms	Completed	NCT00189345
Catumaxomab	Trifunctional IgG type antibody	Neovii Biotech	EpCAM, CD3	Phase II/Phase III	Malignant ascites EpCam positive tumors	Completed	NCT00836654
Catumaxomab	Trifunctional IgG type antibody	Neovii Biotech	EpCAM, CD3	Phase II	Gastric Adenocarcinoma after neoadjuvant chemotherapy and curative resection	Completed	NCT00464893
Catumaxomab	Trifunctional IgG type antibody	Neovii Biotech	EpCAM, CD3	Phase II	Gastric Adenocarcinoma after curative resection	Completed	NCT00352833
MT110	BITE	Amgen Research (Munich)	EpCAM, CD3	Phase I	Gastric Cancer or Adenocarcinoma of the Gastro-esophageal Junction, Colorectal Cancer, Breast Cancer, Hormone-Refractory Prostate Cancer, Ovarian Cancer	Completed	NCT00635596
GD2Bi-aATC	T cells preloaded with IgG type bispecific antibody	Barbara Ann Karmanos Cancer Institute	GD2	Phase I/ II	Desmoplastic small round cell tumor, Disseminated neuroblastoma, Metastatic Osteosarcoma, Recurrent Neuroblastoma, Recurrent Osteosarcoma	Recruiting	NCT02173093
MGD007	DART	MacroGenics	gpA33, CD3	Phase I	Relapsed/Refractory Metastatic Colorectal Cancer	Recruiting	NCT02248805
MGD007 and MGA012	DART	MacroGenics	gpA33, CD3, PD-1	Phase I/ Phase II	Relapsed/ Refractory Metastatic Colon Cancer	Recruiting	NCT03531632
ERY974	IgG4 bispecific T cell-redirecting antibody	Chugai Pharmaceutical	GPC3, CD3	Phase I	Solid Tumors	Recruiting	NCT02748837
MM-111	Bispecific antibody fusion protein	Merrimack Pharmaceuticals	HER2	Phase I	HER2 Amplified Solid Tumors Metastatic Breast Cancer	Completed	NCT00911898
MM-111, Herceptin	Bispecific antibody fusion protein	Merrimack Pharmaceuticals	HER2	Phase I	Refractory HER 2 Amplified Heregulin Positive Breast Cancer	Completed	NCT01097460
ZW25	IgG type bispecific antibody	Zymeworks Inc.	HER2	Phase I	Unresectable and/or metastatic HER2 positive cancers	Recruiting	NCT02892123
Her2 BATs, Recombinant IL-2	T cells preloaded with IgG type bispecific antibody	Yi Miao	HER2	Phase I	Her2 Positive Neoplasms of Digestive System	Unknown	NCT02662348
MCLA-128	IgG type bispecific antibody	Merus N.V.	HER2 and HER3	Phase I/Phase II	Malignant solid tumor Breast cancer Gastric cancer Ovarian cancer Endometrial cancer Non- Small cell lung cancer	Recruiting	NCT02912949
HER-2 BATs with Pembrolizumab	T cells preloaded with IgG type bispecific antibody	University of Virginia	HER2 specific antibody armed activated T cell infusions	Phase I/Phase II	Metastatic Breast Cancer	Recruiting	NCT03272334
Anti-CD3 x HER2- BATs	T cells preloaded with IgG type bispecific antibody	Barbara Ann Karmanos Cancer Institute	HER2, CD3	Phase II	Metastatic Castration Resistant Prostate Cancer	Recruiting	NCT03406858
GBR1302	BEAT-bispecific antibody with heavy chain, light chain and Fc-scFv	Glenmark Pharmaceuticals S.A.	HER2, CD3	Phase I	HER2 Expressing Solid Tumors	Recruiting	NCT02829372
HER2- BATs, Pembrolizumab	T cells preloaded with IgG type bispecific antibody	University of Virginia	HER2, CD3, PD-1	Phase I/ Phase II	Metastatic Breast Cancer	Recruiting	NCT03272334
MCLA-128/ trastuzumab/chemotherapy, MCLA-128, endocrine therapy	IgG type bispecific antibody	Merus N.V.	HER2, ER	Phase II	Metastatic Breast Cancer	Not yet recruiting	NCT03321981
IMCgp100	TCR fused to ScFv	Immunocore Ltd.	HLA A2, CD3	Phase I	Advanced Malignant Melanoma	Completed	NCT01211262
FS118	IgG type bispecific antibody with Fc capable of antigen binding	F-star Delta Limited	LAG3, PD-L1	Phase I	Advanced Malignancies	Recruiting	NCT03440437
LY3164530	IgG4 antibody combined with scFV	Eli Lilly and Company	MET and Anti- EGFR	Phase I	Metastatic neoplasm including NSCLC	Completed	NCT02221882
Anti-CD3-MUC1 and Activated CIK	Fusion of two Fab fragments	Benhealth Biopharmaceutical (Shenzhen) Co., Ltd	MUC1, CD3	Phase II	Advanced liver cancer	Recruiting	NCT03146637
Activated CIK with CD3-MUC1	Fusion of two Fab fragments	Fuda Cancer Hospital, Guangzhou	MUC1, CD3	Phase II	Advanced liver cancer	Recruiting	NCT03484962
Activated CIK with CD3-MUC1	Fusion of two Fab fragments	Fuda Cancer Hospital, Guangzhou	MUC1, CD3	Phase II	Advanced gastric cancer	Recruiting	NCT03554395
Activated CIK with CD3-MUC1	Fusion of two Fab fragments	Fuda Cancer Hospital, Guangzhou	MUC1, CD3	Phase II	Advanced kidney cancer	Recruiting	NCT03540199
Activated CIK with CD3- MUC1	Fusion of two Fab fragments	Fuda Cancer Hospital, Guangzhou	MUC1, CD3	Phase II	Advanced breast cancer	Recruiting	NCT03524261
Activated CIK with CD3- MUC1	Fusion of two Fab fragments	Fuda Cancer Hospital, Guangzhou	MUC1, CD3	Phase II	Advanced lung cancer	Recruiting	NCT03501056
Activated CIK with CD3-MUC1	Fusion of two Fab fragments	Fuda Cancer Hospital, Guangzhou	MUC1, CD3	Phase II	Advanced colorectal cancer	Recruiting	NCT03524274
Activated CIK with CD3-MUC1	Fusion of two Fab fragments	Fuda Cancer Hospital, Guangzhou	MUC1, CD3	Phase II	Advanced pancreatic cancer	Recruiting	NCT03509298
PF-06671008	DART	Pfizer	P-Cadherin, CD3	Phase I	Advanced Solid Tumors	Recruiting	NCT02659631
XmAb20717	Fc engineered bispecific antibody	Xencor	PD1, CTLA4	Phase I	Selected Advanced Solid Tumors	Recruiting	NCT03517488
MGD013	DART	MacroGenics	PDL-1, LAG-3	Phase I	Unresectable or Metastatic Neoplasm	Recruiting	NCT03219268
ES414	scFv domains linked to Fc of IgG1	Aptevo Therapeutics	PSMA, CD3	Phase I	Metastatic Castration Resistant Prostate Cancer	Recruiting	NCT02262910
BAY2010112	BITE	Bayer	PSMA, CD3	Phase I	Castration Resistant Prostate Cancer	Active, not recruiting	NCT01723475
XmAb18087	Fc engineered bispecific antibody	Xencor	SSTR2, CD3	Phase I	Advanced Neuroendocrine Tumor and Gastrointestinal Stromal Tumors	Recruiting	NCT03411915

### Radioimmunotherapy

Radioimmunoscintigraphy (RIS) and radioimmunotherapy (RIT) use the antibody specificity of tumor-based antigens in conjunction with emitted radiation from suitable radioisotopes to image malignancies (RIS) for diagnostic and treatment purposes. The radioantibody is injected intravenously and distributes to the antigen binding site on tumor cells where the radionuclide delivers the tumoricidal dose to the tumor mass (Larson et al. 2015)**.** RIT allows the delivery of radiation doses to multiple dispersed sites simultaneously which makes it effective in killing tumors that have already metastasized to multiple organs (Yang et al. 2016)**.** RIT has traditionally proven efficacious in hematological malignancies such as non-Hodgkin’s lymphoma, which are characterized as more radiosensitive than solid tumor malignancies (Song and Sgouros 2011)**.** Solid tumor malignancies require higher radiation doses to induce tumor cell apoptosis leading to increased adverse effects to radiosensitive areas such as the kidney, lung, and bone marrow. However, intra-compartmental RIT, the addition of chemotherapy to RIT, and the use of alpha emitters are being used to improve the therapeutic index of RIT in solid tumors with less toxicity (Larson et al. 2015)**.** One limitation of conventional radioimmunotherapy is the prolonged radiation exposure of nontarget normal tissues and poor tumor to normal tissue radiation absorbed dose ratios. The development of pre-targeted radioimmunotherapy (PRIT), which first allows tumor specific antibodies to distribute to the tumor antigen site followed by the administration of a small radioactive agent with high affinity for the tumor antibody, limits the accumulation of radiation in non-target sites (Larson et al. 2015)**.** Several bispecific antibodies have been designed and used in these capacities as pre-targets for RIT in addition to providing high specificity binding sites for both the tumor antigen and the radioactive material promoting tumor cell death. A trivalent bispecific antibody, TF12, which targets epithelial glycoprotein − 1 antigen (EGP-1 or TROP-2) and histamine- succinyl-glycine (HSG), when used with lutetium-177 labeled peptide for PRIT in prostate cancer demonstrated effective targeting and permeability in mice pre-clinical studies (van Rij et al. 2014). There are many ongoing clinical trials examining the role of bispecific antibodies in RIT for solid tumors eg. colorectal carcinoma [NCT00185081], [NCT02587247], [NCT000860860], HER2 negative breast cancer [NCT01730612], and lung cancer [NCT01221675] (No Title. clinicaltrials.gov n.d.).

### Targeting carcino-embryonic antigen (CEA)

Trials are studying the bi-specific targeting of CD3 in combination with the tumor antigen, CEA, which is highly expressed in gastrointestinal malignances, non-small cell lung cancer (NSCLC), breast cancer, uterine, and bladder cancers [NCT02324257], [NCT01284231] (No Title. clinicaltrials.gov n.d.). CEA is also used as a target in the DNL TF2 antibody which divalently binds CEA and monovalently binds the peptide-hapten histamine-succinyl-glycine (HSG). Similar to the TF12 format described above, the TF2 antibody first binds to CEA, then an HSG peptide carrying a radionuclide is given which can be used for imaging of the tumor or to deliver radiation to the tumor cells [NCT00895323], [NCT01730612], [NCT01221675] (No Title. clinicaltrials.gov n.d.). In a recent study comparing pre-targeted immunoPET, which uses TF2 and Ga-IMP-288, to conventional ^18^FDG PET, immunoPET was more sensitive than ^18^FDG PET (67% versus 31%) for detection of colonic liver lesions in an orthotopic murine model (Foubert et al. 2018).

### Targeting MET and EGFR

The receptor tyrosine kinase, MET, is one of the most commonly dysregulated oncogenes in non-small cell lung cancer (NSCLC) and met gene amplification has been shown to be a major mechanism in which cancers develop resistance to EGFR inhibitors (Bean et al. 2007). Phase III studies which solely targeted MET have failed to show clinical benefit in NSCLC (Baldacci et al. 2017) but dual targeting of MET and EGFR using bispecific antibodies is promising (Tang et al. 2008; Castoldi et al. 2013) and is currently being explored in NSCLC [NCT02609776] (No Title. clinicaltrials.gov n.d.) and other solid tumors [NCT02221882] (No Title. clinicaltrials.gov n.d.). However, other studies such as the MEHGAN study have provided definitive clinical evidence refuting this hypothesis (Fayette et al. 2016).

EGFRvIII, a rearranged variant of EGFR frequently expressed and associated with poor prognosis in Glioblastoma Multiforme (GBM), and also found in breast and lung carcinoma, was recently found to be expressed in glioma stem cell lines (Emlet et al. 2014). In vivo, a bi-scFv binding CD3 and EGFRvIII, has shown efficacy, specificity, and potency in mice with established EGFRvIII intra-cerebral tumors and may be promising in this highly fatal and difficult to treat malignancy (Gedeon et al. 2013). Another novel method of targeting EGFR, designed by Engeneic, are bacteria derived nanocells coated with bispecific antibodies which can be packaged with chemotherapy such as doxorubicin or mRNA. The bispecific antibody coating allows specificity to certain tumor antigens such as EGFR and limits toxicity to normal cells. The chemotherapy or mRNA payload is released directly into the tumor cell increasing the potency of the therapeutic agent (Shah et al. 2016)**.**There are several phase I trials examining the efficacy of these molecules in EGFR positive CNS tumors as well as solid tumors [NCT02766699, NCT02687386] (No Title. clinicaltrials.gov n.d.).

### Targeting EpCAM

In addition to catumaxomab described above, many other molecules targeting EpCAM have been explored in clinical trials. In vitro studies showed that Solitomab (MT110), which binds CD3 and targets EpCAM, increased the sensitivity of tumor cells to cytotoxic T cell death in multiple EpCAM positive ovarian and endometrial cancer cell lines including ovarian carcinosarcoma and primary uterine serous papillary carcinoma (Bellone et al. 2016; English et al. 2014). An open- label multi-center dose escalation phase I study was completed in 2015 but the results have not yet been published [NCT00635596] (No Title. clinicaltrials.gov n.d.).

### Targeting the HER 2

Ertumaxomab, a trifunctional bispecific targeting HER/neu, CD3, and Fc receptors showed radiographically confirmed clinical response and safety in phase I trials with metastatic breast cancer patients and promising early results of phase II (Haense et al. 2016; View et al. 2008). The phase II clinical trial however was terminated by the sponsor for financial reasons. A phase I/II trial is currently recruiting patients to study the potential effects of MCLA-128 which bi-specifically targets HER2 and HER3 in HER 2 positive breast cancer and other malignant solid tumors [NCT02912949] (No Title. clinicaltrials.gov n.d.). Another phase I trial is evaluating the bispecific targeting of the extracellular domain of HER 2 in HER 2 positive breast cancer with the humanized antibody ZW25 [NCT02892123] (No Title. clinicaltrials.gov n.d.). While HER2 has already proven to be an effective target in breast cancer, preclinical studies have suggested that targeting HER2 and CD3 T cells in metastatic castrate resistant prostate cancer might be an effective strategy (Vaishampayan et al. 2015).

### Targeting Mucin-1 (MUC-1)

Muc-1 is a membrane protein found on the surface of many adenocarcinomas and plays a role in inhibiting the p53 tumor suppression gene (Wei et al. 2007; Kwak et al. 2010)**.** Previously monoclonal antibody therapy directed at MUC-1 from normal tissues was ineffective but recent advancements in targeting tumor Muc1 has been promising in monoclonal antibody studies and CAR-T cell directed therapy (Danielczyk et al. 2006; Posey et al. 2016). Bispecific targeting of MUC-1 and CD16 to mediate NK cytotoxicity cells to target tumor cells in xenograft models has shown effective tumor suppression (Li et al. 2018). A novel PD-1 inhibitor induced cytokine- induced killer cells (CIKs) mixed with an anti-MUC 1 and anti- CD3 bispecific antibody is currently being investigated in several clinical trials for the treatment of advanced solid tumor malignancies [NCT03540199], [NCT03524261], [NCT03501056], NCT03524274], [NCT03509298] (No Title. clinicaltrials.gov n.d.). The bispecific antibody binds to CD3 on the CIK and MUC1 on tumor cells, crosslinking the CIKs and tumor cells promoting effective tumor cell lysis.

### Targeting the prostate-specific membrane antigen (PSMA)

PSMA expressed predominantly in prostate cancer cells as well as the neovasculature of most solid tumors has been a common target in prostate cancer patients (Rajasekaran 2005). A bispecific diabody targeting PSMA in conjunction with antibodies against CD3 has decreased prostate specific antigen levels, inhibited tumor growth and prolonged survival in preclinical mouse studies (Bühler et al. 2008).

### Targeting the immune checkpoint molecules

An interesting strategy of simultaneous blockade of immune checkpoint molecules such as cytotoxic T-lymphocyte antigen-4 (CTLA4) or programmed death-1 (PD-1) or its ligand (PD-L1) and transforming growth factor-β (TGF-β), which mediates immune tolerance using a bifunctional antibody-ligand trap was recently reported (Ravi et al. 2018). One such novel DART bispecific antibody, FS118, which simultaneously inhibits two checkpoint molecules, PDL-1 and lymphocyte activation gene 3 protein (LAG3) is currently in phase I trial in patients with advanced solid tumor malignancies [NCT03440437] (No Title. clinicaltrials.gov n.d.). These agents may potentially improve the efficacy of checkpoint inhibition in various solid tumors which has traditionally been limited with monotherapy.

### Bi and tri-specific T cell and natural killer cell engagers

Innovative techniques to harness natural killer cell in immunotherapy have introduced the concept of bi-specific killer cell engagers (BiKEs) and tri-specific killer cell engagers (TriKEs). BiKEs are created by the fusion of a single chain variable fragment (Fv) against CD 16 (antigen on natural killer cells) and a single-chain Fv against a tumor associated antigen (Rezvani and Rouce 2015). TriKEs are a combination of a single-chain Fv against CD16 and two tumor associated antigens. These molecules directly trigger NK cell activation through CD 16 amplifying NK cell cytolytic activity and cytokine production against various tumor cell antigen targets (Gleason et al. 2012). These drugs are currently being investigated in preclinical studies and safety remains a concern with the potential to trigger cytokine cascades (Tay et al. 2016).

### Targeting other tumor associated antigens

Other tumor-associated antigens that have been studied in monoclonal antibody directed therapy are currently being investigated for bi-specific therapy. Some of these promising target antigens are not just tumor markers but molecules found to be essential in tumor cell survival and proliferation. For example, a bi-specific antibody that targets melanoma- associated chondroitin sulfate proteoglycan (MCSP) and targets the death receptor 5 (DR5, TRAIL-R2), can selectively and potently kill melanoma cells. This approach may prove beneficial in those patients resistant to monoclonal antibody therapy (He et al. 2016). rM28, a single chain bispecific format also targeting a melanoma associated proteoglycan recombinant, in addition to targeting CD28, a co-stimulatory molecule on T cells, showed tumor cell killing without the need for additional TCR/ CD3 stimulation (Grosse-Hovest et al. 2003). P- Cadherin, a cell-to cell adhesion molecule, is upregulated in various solid tumors and overexpression is associated with poor prognosis in breast, ovarian, endometrial, colorectal cancers, intrahepatic cholangiocarcinoma, and pancreatic cancer. Pre-clinical studies of DART antibodies targeting P- Cadherin and CD3 showed significant regression of solid tumors in vitro and in vivo in mice (Fisher et al. 2018). Pfizer is currently conducting a dose escalation study of this molecule, PF-06671008, in patients with P-Cadherin expressing NSCLC, CRC, and triple negative breast cancer (TNBC) [NCT02659631] (No Title. clinicaltrials.gov n.d.). Vascular endothelial growth factor (VEGF) and Angiopoietin-2 (Ang-2), both essential in tumor angiogenesis and escape, are being studied in various combination bispecific formats [NCT03035253], [NCT03292783], [NCT02665416], NCT03030287] (No Title. clinicaltrials.gov n.d.).

### Simultaneous targeting of multiple antigens

Even though most polyspecific antibodies have two binding sites (bispecific), there are many new molecules with three or four binding sites. For example, Castoldi et al.*,* have recently developed a tetravalent Fc containing antibody (tetramab) directed against HER1, HER3, c-MET and IGF1R with enhanced antitumor effects in a preclinical model (Castoldi et al. 2016).

### Other novel applications of PsMab therapy

**Fig. 3:**
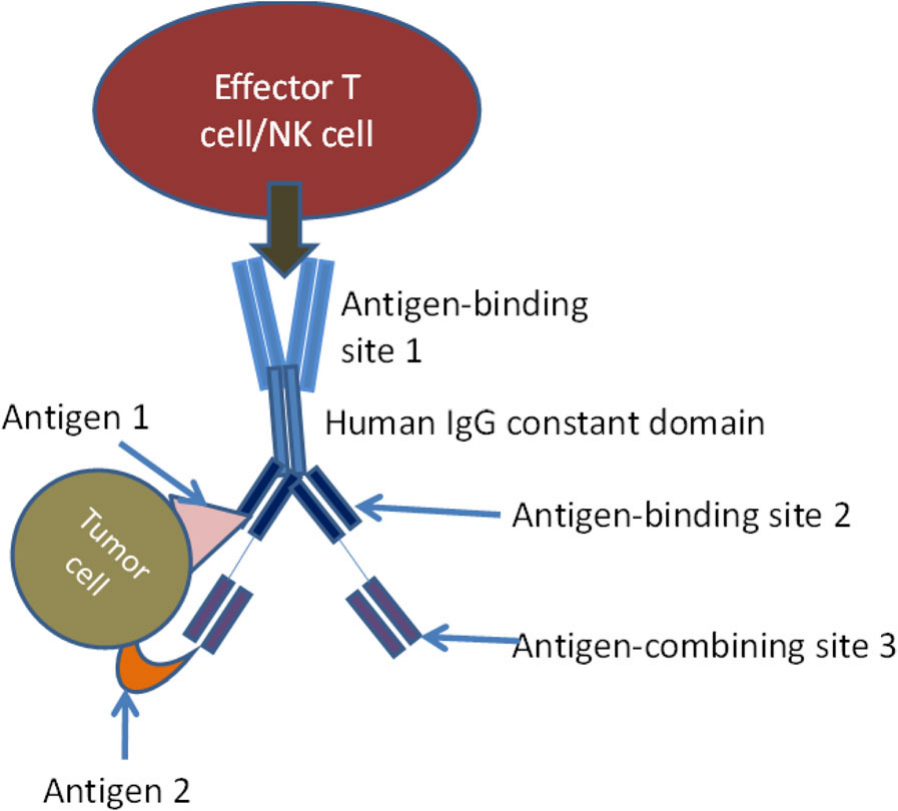
Trispecific antibody. Usually created by using variable domain genes if specific monoclonal antibodies. In this case, antigen-binding site 1 is bivalent and engages with an antigen on effector cell, antigen-binding sites 2 and 3 engage antigens 1 and 2 on tumor cells

Another example of how novel approaches may simplify treatment is a recent preclinical study demonstrating elimination of large tumors by in-vivo production of bispecific antibodies induced by parenterally administered engineered mRNA (Holzinger et al. 2016). If applicable to humans, the high cost of immunotherapy could be dropped to levels of more traditional agents. Another intriguing approach to enhance antitumor effect is the use of a tri-specific antibody with an IL-15 cross linker causing enhanced NK activity antitumor activity (Schmohl et al. 2016). If the construct turns out to be non- immunogenic and has enough drug like properties to allow it to advance in development, the agent may offer an attractive treatment for appropriate tumors. The structure of a typical tri-specific antibody is shown in (Fig. 3).

#### Chimeric antigen receptor adoptive T cell therapy (CAR-T)

Advances in antibody directed therapy have simultaneously fostered the development of another form of immunotherapy, CAR-T cell therapy. While an in-depth discussion on this exciting topic is out of scope for this review article which is focused on polyspecific antibodies, we present a brief review on the topic here and compare the two forms of immunotherapy. CAR-T cell therapy consists on removing T cells from patients and modifying ex vivo using gene transfer to enable expression of specific receptors targeting tumor cells through an antibody-derived binding domain. Once the T cells are genetically modified to express the chimeric antigen receptor, they are infused back into the patients to directly kill the cancer cells (Fig. 2) (Caruana et al. 2014). Currently, CAR T-cell therapy has demonstrated significant anti-tumor activity in the treatment of hematological malignancies. Tisagenlecleucel, CAR T-cell therapy directed at CD19 B- cells, was FDA approved in August 2017 for treatment of pediatric patients with refractory or relapsed B-cell precursor acute lymphoblastic leukemia (Mullard 2017). Multiple CAR-T agents are currently in advanced stages of clinical development for various hematological malignancies (Gauthier and Yakoub-Agha 2017). In contrast, the success of CAR T-cell therapy in solid tumors however has been limited due to the complex tumor microenvironment and difficulty finding suitable target antigens (Gauthier and Yakoub-Agha 2017; Zeltsman et al. 2017). Toxicity and cost have also been major issues with CAR-T cell therapy that are being actively discussed in various forums (Gauthier and Yakoub-Agha 2017; Abbasi 2017). Polyspecific antibodies may offer advantages over CAR-T cell therapy in a multitude of ways and the major differences between the two strategies are tabulated in (Table 5).

**Table 5:** Comparing bi-specific and tri-specific antibody therapy to CAR T- cell therapy

Polyspecific antibodies	CAR T- cells
Polyspecific are antibodies with multiple specificities with one or more affinity sites towards tumor antigens, and another one towards an activator on immune effectors (e.g. CD3 on T cells).	T cells with genetically engineered receptors that redirect them to a chosen tumor antigen
Highly efficient, rapid process- Created using multiple formats including advanced protein engineering and recombinant DNA technologies and administered to patients directly. Allows rapid treatment of patients	Cumbersome, boutique process- T cells from patients are collected, antigen specific receptors (CARs) are inserted invitro into T cells using viral vectors, DNA transposons, or RNA transfection and then “expanded” in the laboratory before reinfusing into lyphodepleted patient. May delay therapy for weeks.
When activated through CD3, cytotoxic T cells inject perforin and granzyme B into target cells to kill.	When CARs bind to tumor antigen, the intracellular signaling domain is activated and the tumoricidal process by T cells is initiated.

While both monoclonal antibody therapy and CAR T-cells are antigen specific immunotherapies, CAR T cells, at present, have to be individually manufactured for each patient resulting in high cost of production. Despite this, CAR T-cells exhibit several qualities that could make them more advantageous than antibody directed tumor therapy. The genetically engineered receptors allow CAR T-cells to recognize tumor cells with low antigen expression and cause direct lysis of tumor cells whereas classical monoclonal antibodies need a high density of tumor antigens to trigger the ADCC or complement cascade (Caruana et al. 2014; Gauthier and Yakoub-Agha 2017). The new constructs of polyspecific antibodies also allow enhanced binding to target and in preclinical models have significantly enhanced anticancer activity compared to monoclonal antibodies (Mazor et al. 2017). Another significant advantage of CAR-T cells therapy is that T cells can naturally extravasate and travel between endothelial barriers within tissue, unlike monoclonal antibodies, which are limited by their size. While the older bispecific constructs have the same disadvantage as mAbs, the newer formats can be made smaller than a classical immunoglobulin allowing better distribution but then usually have a reduced plasma half-life (Brinkmann and Kontermann 2017). In terms of duration of activity, limited data on genetically engineered CAR-T cells suggest that these cells may be able to expand in vivo and result in prolonged response to treatment if a memory pool is established (Caruana et al. 2014). Whether this is a long term advantage is unclear as tumor recurrence lacking the target epitope has been noted in hematological studies. On the contrary, the short half-life of most engineered antibodies necessitates multiple doses to ensure effective therapy but this has been also improvised in some of the newer constructs by adding a FC component or chemically modifying the molecule.

## Conclusions

Antibody-based cancer directed therapy is an exciting and rapidly advancing field. The introduction of monoclonal antibodies such as rituximab revolutionized cancer therapy and have given way to the creation of bi-specific and tri-specific antibodies which work with more precision and efficacy than their predecessors. The one currently approved bi-specific antibody therapy, blinatumomab and catumaxomab, have shown improved survival rates and quality of life for subsets of cancer patients. Multiple agents are currently being evaluated in clinical trials while optimal structures and treatment algorithms are being defined to maximize benefit-risk ratio. Newer approaches concurrently targeting checkpoint molecules and cancer-specific antigens seem promising in preclinical models and may change the landscape of cancer therapeutics (Junttila et al. 2014; Hong et al. 2018).

Several biological parameters are still missing in the understanding of the tumor biology and its complex microenvironment. Engineered polyspecific antibodies will likely play a major role in oncotherapeutics as cancer research continues to climb to new heights.

## Abbreviations

ADCC: Antibody-Dependent Cell-mediated Cytotoxicity
CA: Cancer Antigen
CAR: Chimeric Antigen Receptor
CD: Cluster of Differentiation
CEA: CarcinoEmbryonic Antigen
DNA: Deoxyribonucleic Acid
DR: Death Receptor
EGF: Epidermal Growth Factor
EGFR: Epidermal Growth Factor Receptor
FC: Fragment Crystallizable
FDA: Food and Drug Administration
HER: Human Epithelial Growth Factor Receptor
HLA: Human Leukocyte Antigen
IGF1R: Insulin Growth Factor 1 Receptor
IL: InterLeukin
MET: Mesenchymal-Epithelial Transition factor
mRNA: Messenger Ribonucleic Acid
NCT: National Clinical Trial
NK: Natural Killer
TRAIL-R: Tumor Necrosis Factor-Related Apoptosis-inducing Ligand Receptors
